# Trends of new-onset psychosis or mania in psychiatric emergency departments during the COVID19 pandemic: a longitudinal comparative study

**DOI:** 10.1038/s41598-021-00310-w

**Published:** 2021-10-25

**Authors:** Aviv Segev, Efrat Hirsch-Klein, Gershon Kotz, Shiri Kamhi-Nesher, Shikma Halimi, Khalil Qashu, Ephraim Schreiber, Amir Krivoy

**Affiliations:** 1 Shalvata Mental Health Center, Alyat Hanoar 13th St., Hod Hasharon, Israel; 2 Sackler Faculty of Medicine, Tel Aviv University, Tel Aviv, Israel; 3 Department of Psychosis, Institute of Psychiatry, Psychology and Neuroscience (IoPPN), King’s College London, 16 De Crespigny Park, London, SE5 8AF UK; 4 Be’er Yaakov Mental Health Center, 1st Rabin Av., Be’er Ya’akov, Israel; 5 Geha Mental Health Center, 1st Helsinki St., Petach Tikva, Israel

**Keywords:** Psychosis, Viral infection

## Abstract

COVID19 infection was associated with possible psychiatric manifestations, including psychosis and mania. In addition, psychiatric disorders might be triggered by severe psychological reactions to the pandemic or the measures taken to contain it. This study aimed to assess the trends of new-onset psychosis/mania during the pandemic timeline. Psychiatric emergency department records during January-July 2019 and 2020 of two regional mental health centers were manually examined. Cases of new-onset psychosis or mania were found in 326 out of 5161 records examined. The ratio of these cases increased by 45.5% in 2020 compared to 2019 (189 out of 2367, 137 out of 2479, respectively, *p* = 0.001). The peak increase was in April 2020 (9.4% vs. 4.7%, *p* = 0.015). There was no association between the rise of new-onset psychotic or manic episodes and national incidence of COVID19 cases, as observed during Israel 2nd wave. PCR tests were negative, except a single case. In this study, an increase in new-onset psychosis/mania was identified during the initial phase of the pandemic. Though causality could not be directly inferred, lack of infection symptoms, negative PCR testing and temporal distribution incongruent with COVID19 caseload did not support a direct effect of SARS-CoV-2. Alternative explanations are discussed, such as psychological reaction to stress and preventive measures, as well as case-shifting between different mental health settings.

## Introduction

The impact of the COVID19 pandemic is being extensively studied since the early stages of its outbreak around January 2020. Much emphasis has been given to the acute stages of the disease, with its respiratory and circulatory complications. However, the Coronavirus family has been suspected before as having the capability to cause neuro-psychiatric symptoms^1^. Several reports have suggested that the severe Acute Respiratory Syndrome Coronavirus-2 (SARS-COV-2) virus is eliciting various neuro-psychiatric symptoms, with unusual proportion at younger ages^2,3^. Reports have shown an increase in non-specific neurological outcomes (e.g. strokes and epilepsy) along with non-specific findings on neuroimaging^4^. Possible mechanisms that have been suggested are micro-occlusions, inflammatory or immunological response and direct damage of the virus. Several cases of new-onset psychosis of patients with a positive polymerase chain reaction (PCR) test for SARS-CoV-2 were reported^5–7^. One case study presented an hyper-intense signal of the olfactory bulb and right gyrus rectus followed by thinning of the olfactory bulb in an MRI scan of a patient with positive PCR result for SARS-CoV-2^8^.

In addition to those notions, others suggested that the overall sensation of panic and stress due to the pandemic, in combination with the social and economic consequences of quarantine might give rise to negative psychiatric outcomes^9–11^. Increased prevalence of depression, post-traumatic stress disorder and other mental health consequences has been documented, with excess risk at certain groups (lower economic status, female sex, lower social support and more)^9^. These trends were also observed in previous epidemics^9^. Supporting that, Increased prevalence of “reactive psychosis” was thought to be related to the extreme social changes (lockdowns, unemployment, social distancing)^12,13^. Furthermore, a correlation between the severity of quarantine conditions (e.g. isolation) and psychotic symptoms was described^14^.

However, these are all case reports and could be incidental or highly unusual occurrences, when studying the effects of a highly infectious disease already inflicting over 150 million people worldwide.

Studies examining trends of psychiatric referrals and emergency visits found an overall decrease in the number of presentations^15–17^. A relative increase in psychotic and manic presentation was observed in some studies, as well as increased severity, manifested in greater numbers of involuntary admissions^15,17,18^. Other studies, however, report similar or slight decrease in psychotic cases^16,19^.While studies acknowledged the different pathways in which the pandemic can affect these trends (biological vs psychological and sociological), results were conflicting, whether COVID19 positive patients were more likely to be psychotic^19^, or no difference was observed between COVID19 positive and negative patients^15^.

The aim of this study is to examine whether the COVID19 pandemic has led to an increase in the number of new-onset severe psychiatric manifestations, specifically psychoses and manic episodes.

## Material and methods

### Study population

All patients aged 18 and above, visiting the psychiatric emergency departments (EDs) of two adjacent major mental health centers (Geha, Petach-Tikva and Shalvata, Hod Hasharon, Israel), between January 1st to July 30th in 2019 and 2020. These two psychiatric centers cover adjacent catchment areas, comprising of 1.5 million people (15% of Israel's population).

The study was approved by both Geha and Shalvata mental health centers’ Institutional Review Boards (IRBs). All methods were carried out in accordance with relevant guidelines and regulations. Due to the nature of the study (retrospective EHR data), both IRBs approved that informed consent is not required.

### Protocol

Electronic medical records (EMR) of all ED visitations were examined. Each of the records was reviewed by a psychiatrist to determine if the presentation was a case of new-onset psychosis/ mania or not. The decision to combine both psychoses with mania was due to the difficulty to distinguish between those entities in their hyper-acute phases and first-time assessment at a single ED visit. Pooling of the two was expected to increase reliability and validity of classification. It should be noted that ICD codes could not be used to classify the cases, as often the diagnosis filled-in is not conclusive (such as "observation for suspected mental and behavioral disorders"). Determination of new onset was established per previous records as well as the patients' and their families' description of events, and was defined when symptomatology manifested from December 1st of the previous year. This timeframe was chosen as evidence suggested that the COVID19 pandemic was already spreading during December 2019^20,21^, so cases related to COVID19 might have started even then. As the comparison of 2019 and 2020 should be symmetrical, the same timeframe was chosen for 2019. The classifications of the type and the timing of the psychiatric presentation were based on the record of full psychiatric assessment and symptoms as described in the EMR during the ED visit. In cases of ambiguity, the full record of the hospital admission itself (if done) or follow-up visits (if done) was reviewed by two senior clinicians to clarify the vagueness of the initial presentation. In cases where data was missing, no classification was recorded. As the focus of this study was new-onset psychosis and mania presentation, all cases defined as such were further re-validated and confirmed by another senior psychiatrist. Furthermore, to verify reliability of the classification process, 5% of total cases were re-classified by a different reviewer.

To gather information regarding possible association with COVID19 infections, records of new-onset disorders during 2020 were examined for SARS-CoV-2 PCR results, if available, and history of COVID19 symptoms for concurrent illness during initial presentation was examined. To note, per Israeli ministry of health policy, from March 2020 all patients assessed by an ED are also questioned using a designated questionnaire to assess the risk for COVID19 (Supplementary 1).

We have used the Oxford Government Response Stringency Index (OxGR), a measure developed to depict the severity of countermeasures taken by authorities to contain the pandemic^22^, in order to estimate the burden of restrictive preventive measures on the public. This measure cannot attest to the level of stress and anxiety in the general public, but as those are both unavailable and highly individual, the OxGR, ranging from 0 to 100, roughly approximate the magnitude of external stressors.

### Ethical considerations

All methods were carried out in accordance with Israel's relevant guidelines and regulations, per retrospective studies analyzing patients' medical records.

### Statistical analysis

The ratio pf patients with new onset psychosis or mania was calculated as percentage of the patients examined in the ED at the same period. We compared proportion between parallel periods in 2019 and 2020 using chi-square analysis. Continues measures (age) was compared by Mann–Whitney U Test. Analyses were done using IBM SPSS version 25.

## Results

There were 2616 ED visits during the first half of 2019 (median age 41.6, range 18–102, 57.8% males) and 2545 visits during the first half of 2020 (median age 40.5, range 18–96.3, 59.3% males), depicting a decline of 2.7% in the number of total visits, and a younger age of patients (*p* = 0.022) in 2020. Due to lack of information regarding past psychiatric history or current clinical presentation, 5.3% of cases in 2019 and 7% of cases in 2020 could not be classified, therefore 2479 and 2367 cases, respectively, were included in the final analysis. Reliability of classification was tested by reclassifying random 301 cases (5.8% of the total cases) by a different reviewer. Inter-rated agreement was 99.0% and kappa was 0.93.

**Figure 1:**
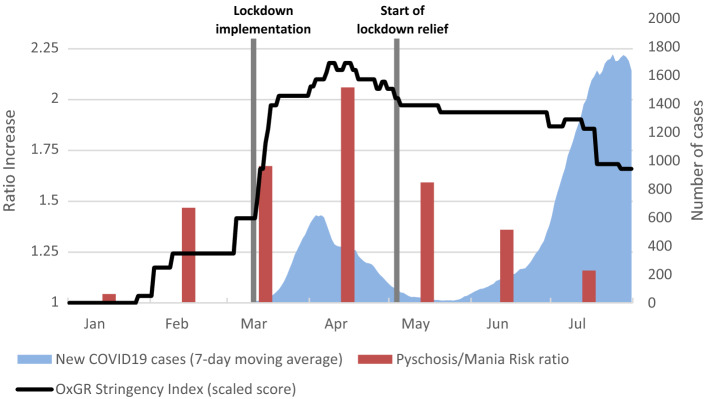
Monthly Risk ratio of new-onset psychosis and mania in 2019 and 2020. OxGR: Oxford Government Response Stringency Index; originally ranging 0–100, Linearly scaled in this graph to 1.00–2.25 to allow proportional presentation with the other two sets of data.

In 2019 there were 137 (5.5%) of new-onset psychosis or mania, compared to 189 (8.0%) in 2020, reflecting an increase of 38.0% in the number of new onset psychosis or mania cases. The ratio of new-onset psychoses/mania had significantly risen by 1.44 (*p* = 0.001). Per month ratio analyses (Table 1) showed a significant sharp rise in April and May. The ratio per month is presented in Fig. 1, along with the timeline of incidence of COVID19 cases in Israel ^23,24^ and Oxford University Government Response (OxGR) Stringency Index, depicting the severity of countermeasures taken by authorities to contain the pandemic^22^. The OxGR is based on data collected from publicly available information by a cross-disciplinary Oxford University team, that integrates policies such as school closures, travel bans, etc. These indicators are transformed into a composite measure ranging from 0 to 100, and is meant for comparative purposes.

**Table 1:** Risk Ratio of new-onset psychoses or mania by month in 2019 and 2020.

Month	2019 N (rate)	2020 N (rate)	risk ratio	Confidence interval	*P* value
January	18/390 (4.6%)	22/456 (4.8%)	1.05	0.57–1.92	> 0.1
February	17/365 (4.7%)	25/364 (6.9%)	1.47	0.81–2.68	> 0.1
March	20/384 (5.2%)	27/311 (8.7%)	1.67	0.95–2.91	0.07
April	16/323 (5.0%)	25/243 (10.3%)	2.08	1.13–3.80	0.015
May	28/345 (8.1%)	44/342 (12.9%)	1.59	1.01–2.49	0.042
June	17/341 (5.0%)	21/310 (6.8%)	1.36	0.73–2.53	> 0.1
July	21/331 (6.3%)	25/341 (7.3%)	1.16	0.66–2.02	> 0.1
Total	137/2479 (5.5%)	189/2367 (8.0%)	1.44	1.17–1.79	= 0.001

As for the timeline, the year began with a slight non-significant rise in the number of cases compared to 2019. This has peaked till April and May and declined through June and a further in July. This pattern seems to precede the 1st wave of COVID19 morbidity in Israel. There was no increase in new-onset psychoses and mania during Israel’s 2nd wave (June-July), which entailed heavier burden of cases, morbidity and mortality than the 1st wave (March–April). The dramatic increase in COVID19 cases during June (7.3-fold) and July (40.6-fold), was not accompanied by a concordant increase and even absolute and relative decrease in new cases of psychosis/mania, to a level even lower than January.

The new-onset psychoses and mania groups did not differ in gender distribution between 2019 and 2020, however the mean age of these patients showed a borderline significant difference in age, as patients in 2020 were younger compared to 2019 (median[IQR] = 29.0[18.79] and 34.75[21.75], respectively, *p* = 0.03).

Of the patients presenting with new-onset psychosis or mania during the time in which PCR testing was available (1.4.20–31.7.20), 73 patients out of 115 (63.5%) were tested for SARS-COV-2 by PCR on admission. Only one test was positive. The others were not tested as policy regarding PCR tests prior to admissions had changed during the period. Out of 112, 95.5% of the patients (n = 107) were administered a COVID19 questionnaire at the during the ED visit (supplementary 1). Additional 77 (40.7%) patients developed psychosis during January-March, when the questionnaire was not yet administered. Out of patients filling symptom questionnaire, 12 reported recent symptoms suggestive of COVID19 (cough and malaise, without fever), 8 of those had a negative PCR test (4 were not tested). Additional three reported either a visit to a hospital or a contact with a person with fever within the last 14 days. Two of them were found negative in a PCR test (the third was not tested). One patient, admitted for new-onset psychosis, was suspected to have encephalopathy due to abnormal EEG results. Lumbar puncture and MRI supported an organic process, however several PCR tests for SARS-COV-2 were negative.

## Discussion

Our results show a significant increase in the rate of patients presenting to the psychiatric emergency departments with new-onset psychoses or manic episodes from January to July 2020 compared to 2019. This rise was not just proportional (as the number of over-all ED visits declined in 2020, congruent to other reports^15–17^), but was also shown in absolute numbers. While other studies reported an increase in the number of people showing with psychotic presentations^15,17,18^, our study has shown even greater increase in the rate of new-onset episodes. Moreover, patients presenting with new-onset psychoses and manic episodes had a younger age of onset during 2020. This finding is congruent with an overall younger age of all patients visiting the ED in 2020, perhaps due to concerns of older people from places perceived as COVID19 high-risk. It should be noted that an opposite trend was previously described^18^.

In almost all cases in our study, present or past COVID19 infection was not suggested by PCR tests, designated symptoms questionnaires, clinical presentation, or medical records. Only a single case was tested Positive for SARS-CoV-2 PCR test.

It should also be mentioned that by the end of July 2020 almost 1% of the population in Israel had been infected (per PCR testing) and serological testing and epidemiological studies suggested that the real number of cases was higher^25^. As such, the ratio of positive PCR tests among the new-onset cases was lower than that of the general population. This might be the result of an additional minimization of social interaction often preceding psychotic episode.

Therefore, our study could not find epidemiological or biological evidence for a causal link between COVID19 caseload and increased ratio of new-onset psychosis.

While lack of concurrent COVID19 infection and psychosis in our data could not rule out the possibility for a biological causation (such as psychosis as a late sequela of COVID19 infection), the absence of temporal correlation of psychosis with COVID19 infection might further support the psycho-social etiology of psychotic or manic manifestation. As mentioned, several case reports and theories suggested that psychological factors might cause an increase in psychiatric exacerbations^9,26,27^. Although the possible effect of stress and isolation is difficult to quantify^14^, as opposed to number of new COVID19 cases, the temporal course and the pandemic timeline might suggest that psychological factors did play a more prominent role in the outcome. The rise in the new-onset cases preceded the actual propagation of the pandemic (first case was discovered in Israel on February 27^th^), while uncertainty and alarming reports came from China and Italy. The peak increase of new-onset psychosis or mania cases coincided with the implementation of preventive restrictive measures in Israel, and especially when a strict lockdown was implemented as off mid-March (school closure, complete cessation of non-essential business, followed by ban on gathering, 500-m from home distance restriction, mandatory face masks including outdoors and controversial authorization for the Israeli security agency to track citizens by cellular signal to improve contact- tracing)^28^. This period was accompanied by financial insecurity and record-high numbers of unemployment^29^ due to strict lockdown, loss of daily routines, disturbed sense of normality, heightened anxiety, loneliness, and an increase in family feud^30^. During May, the lockdown was gradually lifted and realization that COVID19 mortality was overestimated had trickled to the public^31,32^, along with a relatively modest 1st wave that Israel went through (30 deaths per million). All these elements, permeated to the public, might have contributed to a sense of pseudo-normalization of the life in COVID19 world, lessening the initial psychological burden arising with the pandemic emergence^9^. Though the number of COVID19 cases rose up quickly to a level much higher than the 1st wave, the Israeli government did not impose new restrictions and even eased the existing one (until mid-September, when a new lockdown was imposed – outside this study timeframe)^22–24^.

Additional explanation to the findings is as dread arose from the pandemic, patients were more inclined to avoid hospitals, behavior that was reported both in psychiatric^19^ and other fields of medicine^33^. This was shown through the decrease in the total number of ED visits, as well as a decrease in non-new-onset cases. It is possible that patients already registered in mental health service were more inclined to avoid ED visit, and to wait for their responsible psychiatrist, even at the cost of a short delay. However, outpatient services were in-part minimized and many were transformed to tele-psychiatry. As such, it is possible that patients and families dealing with an emerging acute psychiatric disorder for the first time had almost no alternative than to approach a psychiatric ED, since engaging with outpatient clinics became more difficult. This might have caused shifting of milder cases (usually managed at the community services level) to hospital setting, as also shown by reported reduction of new mental health diagnoses in the primary care setting^34^.

### Limitations

We cannot rule out the possibility that 2019 had unusual low ratio of new-onset psychosis and mania. However, if that is the case, it would still point out to the low plausibility of causal biological association between COVID19 and new psychiatric disorder. Our data includes only cases presenting to the ED, therefore generalizability should be done cautiously as new-onset cases who sought treatment in outpatient setting are not depicted. Moreover, this study was done in mental health centers, and it is possible that psychotic and manic episode with atypical features, following COVID19 infections, were more likely to be referred to general hospitals rather than a psychiatric center. Further studies might be able to examine if a similar increase was observed in general EDs, and whether these cases were linked to COVID19. Current studies performed in the setting of general hospital, were congruent to our result^15^. Additional limitation of our study is the classification process, done manually by a psychiatrist. However, access to clinical note, re-verification by a senior psychiatrist and an excellent kappa value, suggest that bias is unlikely to distort the result. Last, the scope of this study could not rule out or prove either biological, psychological, or technical reasons for the surge of new-onset psychosis or manic episodes. Though all biological measures applied did not support a biological association, they cannot rule out an asymptomatic or mild course of COVID19, with late neuro-psychiatric sequala. Several pathogens are known to cause late-onset CNS sequala (e.g., measles causing sub-acute sclerosing pan-encephalitis, or the Spanish flu and encephalitis lethargica). However, the association depicted in case series so far was with a close temporal relationship. Serological test might serve as an important tool to further elucidate this hypothesis, though it has been reported that antibodies may become undetectable within months^35^, thus even this methodology might not provide certainty regarding the biological connection. However, the study has strength in that it includes two regional mental health centers with rather large sample during a significant period of follow-up time.

## Conclusions

There was a significant increase in the number and rate of people with new-onset psychosis or mania referred to the psychiatric ED during the 2020 COVID19 pandemic period in comparison to the parallel period in 2019. However, PCR testing, designated questionnaire and mostly the pandemic timeline, do not suggest SARS-CoV-2 infection as the cause. It is possible that the increased number of cases is attributed to stress stemming from the dread arising from the pandemic, social and economic pressure and especially the restrictive effects of the complete lockdown. Additional explanation could also be a change in the patterns of mental health services distribution and consumption during this period. Further studies need to examine the serology of COVID19 in new-onset cases to be able to detect a possible late psychiatric manifestation of COVID19 CNS infection.

Above all, the results of this study emphasize the importance and the need to augment mental health services deployment and capabilities, even in times of somatic-focused medical crisis. As the ripples of the social and economic sequala of the COVID19 crisis will linger and even intensify, the importance of mental health would probably be even more pronounced in the months to come.

## Supplementary Information

Supplementary Information.

## Data Availability

The data can only be accessed by permitted individuals from within a secure firewall (i.e., the data cannot be sent elsewhere), in the same manner as the authors. For more information, please contact the authors or hanref@clalit.org.il.
