# Supplementary material for: Trends of new-onset psychosis or mania in psychiatric emergency departments during the COVID19 pandemic: a longitudinal comparative study
Source: Sci Rep. 2021 Oct 25;11:21002. doi: 10.1038/s41598-021-00310-w (PMC8546064; doi:10.1038/s41598-021-00310-w)
Supplement: Supplementary file 1 — Supplementary Information. [file 41598_2021_310_MOESM1_ESM.docx]

**Supplementary 1**

**COVID19 Risk Questionnaire***

1. Do you currently, or did you lately, experience:
   1. Fever above 38c
   2. Fever
   3. Cough
   4. Shortness of breath
   5. Cough
   6. Loss of sense of taste
   7. Loss of sense of smell
   8. Other respiratory symptoms (not including rhinorrhea)
   9. Extreme weakness or muscle pain
   10. Sore throat or rhinorrhea
   11. Diarrhea
   12. Other
   13. How many days are you ill?
   14. Were you out of the country in the 14 days preceding the onset of symptoms?
2. Have you returned from overseas in the last 14 days?
3. Are you currently at self-quarantine?
4. Do you work in a place where you are exposed to high traffic of people, or exposed to travelers from overseas in the last 14 days?
5. Do you work in a healthcare facility, care homes for the elderly or disabled, or any other closed therapeutical community?
6. Do you live in a closed community in which a case of COVID19 was detected, especially if high-risk for severe morbidity or crowded?
7. Were you directly exposed to people returning from overseas in the last 14 days (at work, family, etc.)?
8. Did you participate in crowded events?
9. Do you work directly with clients?
10. Were you exposed to patients with fever, or visited a hospital or community clinic?
11. Do you live in a place where a cluster/outbreak was defined?

* This questionnaire was translated from Hebrew.
